# AFD-StackGAN: Automatic Mask Generation Network for Face De-Occlusion Using StackGAN

**DOI:** 10.3390/s22051747

**Published:** 2022-02-23

**Authors:** Abdul Jabbar, Xi Li, Muhammad Assam, Javed Ali Khan, Marwa Obayya, Mimouna Abdullah Alkhonaini, Fahd N. Al-Wesabi, Muhammad Assad

**Affiliations:** 1 College of Computer Science, Zhejiang University, Hangzhou 310027, China; jabbar@zju.edu.cn (A.J.); assam@zju.edu.cn (M.A.); 2 Department of Software Engineering, University of Science and Technology, Bunnu 28100, Pakistan; javed_ali@ustb.edu.pk; 3 Department of Biomedical Engineering, College of Engineering, Princess Nourah Bint Abdulrahman University, Riyadh 11671, Saudi Arabia; ma.obayya@pnu.edu.sa; 4 Department of Computer Science, College of Computer and Information Sciences, Prince Sultan University, Riyadh 12435, Saudi Arabia; mi.alkhonani@psu.edu.sa; 5 Department of Computer Science, College of Science & Art at Mahayil, King Khalid University, Abha 62529, Saudi Arabia; falwesabi@kku.edu; 6 Institute for Frontier Materials, Deakin University, Geelong, VIC 3216, Australia; asadm@deakin.edu.au

**Keywords:** generative adversarial network (GAN), automatic mask removal, image restoration

## Abstract

To address the problem of automatically detecting and removing the mask without user interaction, we present a GAN-based automatic approach for face de-occlusion, called Automatic Mask Generation Network for Face De-occlusion Using Stacked Generative Adversarial Networks (AFD-StackGAN). In this approach, we decompose the problem into two primary stages (i.e., Stage-I Network and Stage-II Network) and employ a separate GAN in both stages. Stage-I Network (Binary Mask Generation Network) automatically creates a binary mask for the masked region in the input images (occluded images). Then, Stage-II Network (Face De-occlusion Network) removes the mask object and synthesizes the damaged region with fine details while retaining the restored face’s appearance and structural consistency. Furthermore, we create a paired synthetic face-occluded dataset using the publicly available CelebA face images to train the proposed model. AFD-StackGAN is evaluated using real-world test images gathered from the Internet. Our extensive experimental results confirm the robustness and efficiency of the proposed model in removing complex mask objects from facial images compared to the previous image manipulation approaches. Additionally, we provide ablation studies for performance comparison between the user-defined mask and auto-defined mask and demonstrate the benefits of refiner networks in the generation process.

## 1. Introduction

Face occlusion, a growing trend in recent years worldwide, is one of the leading causes of computer vision problems, such as face recognition, identification, tracking, detection, classification, face parsing, contour extraction, etc., which are challenging to tackle. Faces play the most substantial role in describing human face characteristics, facial identity, facial expression, and facial emotions. Thus, people used several methods, such as wearing fancy masks, painting the face with makeup, or pasting a tattoo, to hide their face characteristics, identity, expression, and emotions from the public, video surveillance cameras, or face verification systems because content replacement by serious occlusion with non-face objects always produces partial appearance and ambiguous representation. Obtaining high-resolution and non-occluded face images from occluded face images is essential but challenging for face analysis because faces usually contain few repetitive structures. For successful face recognition systems (FRS) or guessing someone’s identity, removing the occulted object covering most of the face and correctly restoring the face’s missing contents without destroying the existing data distribution is very important. The performance of a face recognition system (FRS) model may often degrade in the presence of unknown occlusions or disguises. Removing the mask object covering the human face’s discriminative region and then correctly restoring the face’s missing contents might help guess someone’s face secret identity.

Over the last several years, researchers have made significant progress in creating image synthesis algorithms that turn an occluded face image into an occlusion-free face image. They have achieved promising results for removing an object in an image; however, they feature some unignorable defects associated with the affected regions, such as lack of high-frequency and perceptual information in situations where they have to deal with occlusion masks of large objects of complex nature, and have significant variations in the structure, size, shape, type, and position in the face image. This is primarily because these methods are trained where occlusion masks, including medical masks, sunglasses, eyeglasses, microphones, scarves, cups, hands, and flowers, have less structure, size, shape, type, position variations in the face image. Their algorithms also show severe deformations and aliasing flaws in their results, especially for regions around the eyes. Such degraded results severely affect many computer vision systems, such as recognition, identification, tracking, detection, and classification.

The biggest motivation behind this research is to de-occlude the occluded parts of an image while keeping the image smoothness unaffected, focusing on the facial area, i.e., removal of the self-employed non-face objects/foreground occluding objects which fill the hole left behind in facial images with visually plausible content. This involves the automatic creation of varied binary masks for the occluded regions after detecting them in the input images (occluded images) and then inpainting the holes left behind after removing unwanted objects from images with plausible correct contents and fine detail. Various occlusions regions are observed from actual face images. Thus, automatically, face occlusions pose a challenging task because:

1. The result heavily depends on the accuracy of detection of the occluded region (i.e., failing to detect an occluded region properly may cause generation of poor binary mask that severely affects de-occlusion task);
2. It is not easy to recover complex semantics of the face under the occluded region detected due to significant variations in the occluded region (i.e., occluded objects/non-face items have vast structures, sizes, colors, shapes, types, and positions variations in the facial images);
3. Training data, i.e., facial image pairs with and without mask object datasets, are sparse or non-existent.

The proposed model proposes an interaction-free approach (i.e., the proposed approach can perform face de-occlusion without requiring a manual occlusion mask) that first generates the binary mask for the occluded region of random sizes, shapes, colors, and structures after detecting it and then removes the non–face objects from the foreground of the input occluded facial images while maintaining the face’s overall coherency.

**Figure 1:**
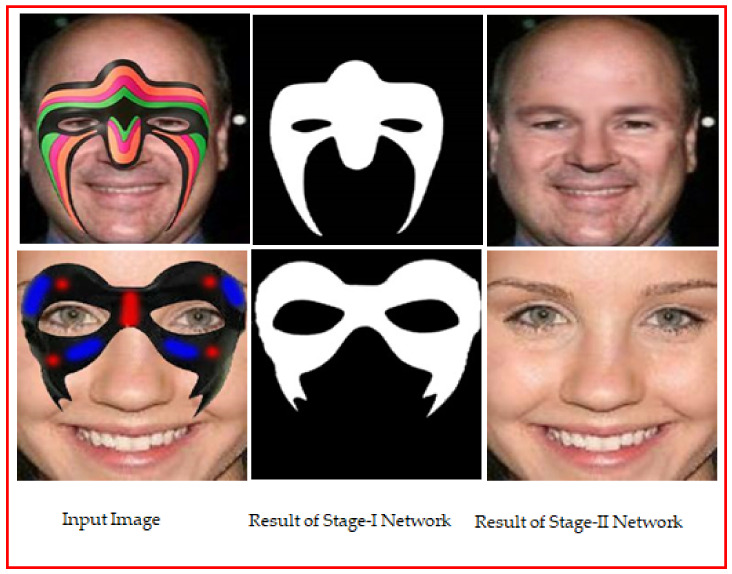
The proposed AFD-StackGAN results on real-world images.

An example result of GAN [1] based automatic mask generation network for face de-occlusion using StackGAN (AFD-StackGAN) is shown in Figure 1. Following the well-known “coarse-to-fine structure recovery method,” the proposed model’s Stage-I Network (Binary Mask Generation Network) generates a binary mask for the masked region after detecting the mask object in the input facial images. Then, Stage-II Network (Face De-occlusion Network) removes the mask object and synthesizes the damaged region with plausible content while retaining the global coherency of the face structure. Furthermore, we trained the proposed model on a synthetically created facial images dataset. Since there are no facial image pairings with or without mask objects, we have created a paired synthetic dataset using the CelebA dataset. We assessed the proposed model on real-world test images containing non-face items with vast structure, size, color, shape, type, and position variations in the facial images gathered from the Internet. We compared the performance of the proposed model with previous face recovery methods. Several experiments illustrate that the proposed AFD-StackGAN outperforms other previous face recovery methods.

The main contributions of an automatic mask removal network for face de-occlusion are summarized as follows:

- This work proposes a novel GAN-based inpainting method by employing an automatic mask generation network for face de-occlusion without human interaction. This work automatically eliminates challenging mask objects from the face and synthesizes the damaged area with fine details while holding the restored face’s appearance and structural consistency;
- This work attempts to alleviate the manual mask selection burden by creating a straightforward method that can intelligently and automatically generate the occluded region’s binary mask in facial images;
- One potential application of an automatic mask generation network could be a video where mask objects continuously conceal the face’s structural semantics;
- We experimentally show that the proposed model with an automatically generated mask is more effective than those with manually generated masks for removing mask objects and generating realistic semantics of face images.

The structure of this research work is as follows. Section 2 reviews the work related to image editing. The proposed approach, as well as the loss function, is described in Section 3. The proposed scheme’s implementation and training details are discussed in Section 4. Results and comparison are argued in Section 5. Section 6 concludes the whole paper.

## 2. Related Works

This section will cover related work concisely in the context of object detection and removal of objects in an image.

### 2.1. Object Detection Methods

Object detection is the process of finding various objects in an image. Face occlusion detection aims to detect the facial region occluded by other objects. The task of object detection becomes even more complicated when their appearance is invisible to other visible objects in the scene.

R-CNN [2], Fast R-CNN [3], Faster R-CNN [4], and Mask R-CNN [5] are convolution neural network (CNN)-based [6] pioneer works that produce state-of-the-art results for numerous object detection; however, they will require many training samples and a lot of computing power. As a result, instead of employing costly approaches for automatically detecting mask objects (non-face objects) in facial images, we use a simple segmentation network that focuses on the mask object in facial images (occluded images).

A fully convolutional neural network (FCN) [7] is a pioneering end-to-end trained network for image segmentation that uses a CNN-based auto-encoder setup. Several variants of FCN, such as [8,9,10], have been proposed to make it more appropriate for image segmentation tasks. Generally, all these approaches use a modified version of the classification network (removing its fully connected layers and replacing them with a typical CNN layer) as an encoder to produce a low-resolution image representation. De-convolution is used for up-sampling to obtain the output size equal to the input image. However, they use different approaches for mapping encoded representation into pixel-wise prediction. U-shaped (U-Net) [11] is a CNN-based encoder-decoder with skip connections used between mirrored layers in the encoder-decoder network architecture. The U-Net-based network is widely used for fast and precise segmentation of images to have better visual and quality results. U-Net’s encoder captures the context in the image using a series of convolution with max-pooling layers, while the decoder up-samples the encoded information using transposed convolution. Moreover, feature maps from the encoder are concatenated to the feature maps of the decoder. The U-Net has vast applications, especially in medical imaging, object detection, biometric recognition, and surveillance systems.

The Segmentor GAN (Se-GAN) [12] model detects the occluded objects in the same image. The Se-GAN segmentor network takes an image and visible area as its input and generates the mask of the whole object that has been occluded. The Se-GAN generator generates the appearance for the object painting’s occluded area by painting the missing pixels. The discriminator of Se-GAN discriminates the generator generated and the actual image regions. Both Se-GAN networks are trained in an adversarial way to generate an object image with invisible regions. Perceptual-GAN (P-GAN) [13] generates ultra-resolved descriptions of small objects for better detection by decreasing the differences between small and large objects. The P-GAN includes a generator that transforms the small objects’ sparse representations to highly super-resolved images that are sufficiently like actual large objects and a perceptual discriminator that differentiates the generator-generated super-resolved representations of small objects from the real through an adversarial loss. In addition to this, the discriminator network boosts the detection performance through an additional perceptual loss.

Similarly, Multi-Task GAN (MT-GAN) [14] used an SRN (super-resolution network) to up-scale the small-scale distorted image into the large-scale clear image for better detection. It consists of a super-resolution network and a multitask network. MT-GAN’s super-resolution network works as a generator, which up-scales the small-scale distorted image into a large-scale clear image. MT-GAN’s multitask network works as a discriminator to discriminate the real higher-resolution images from those generated, predict object categories scores, and further improve the bounding boxes at once. The GAN-based Detection of Objects (GAN-DO) [15] method recently learned an adversarial objective for object detection through training. GAN-DO takes a low-quality image as input for accurate object detection, in contrast with previous methods that take a high-quality image as input. The discriminator learns to differentiate between the output of higher-quality original data from the pre-trained baseline model and the generator’s different-quality output. The generator learns to outsmart the discriminator. The discriminator classifies the generator output of the augmented data as the output of the original data by the baseline model.

Hence, instead of using these expensive algorithms to detect non-face objects in facial images automatically, we employ a simple encoder-decoder network architecture focusing on mask objects. The encoder-decoder network architecture has three convolution layers for the encoder part and three convolutions (transpose convolution) layers for the decoder part.

### 2.2. Object Removal Methods

Another essential application related to this work is object removal, in which the user removes the non-face object from an image and reconstructs the image by filling in the hole left behind with appropriate contents and adequate details so that the reconstructed image looks real. Image editing/inpainting is a common way of performing this task.

Non-learning-based object removal methods [16,17,18,19] erase mask objects from an image and inpaint the affected region by propagating matching pixels from the neighboring areas using an iterative search approach. Criminisi et al. [16] introduced an exemplar-based texture synthesis technique, a unified methodology for generating plausible texture in a specified region. However, it cannot produce good results for synthesizing areas where matching patches are not present in the image. Wang et al. [17] utilized a modified sum of squared differences and normalized cross-correlation to find the most appropriate patch. Artifacts are generated at the borders of removed items, even though they properly remove the object in essential scenarios. Hays and Efros [18] search through millions of scene images for the most similar information to the input sample, then copy and paste that information into the missing pixels in the input sample. These non-learning procedures provide better results, but they rely greatly on the supplied image data. Park et al. [19] eliminate eyeglasses from face images by adjusting the patch priority function in determining the filling order using a regularized factor. Their technique effectively eliminates tiny objects such as eyeglasses, but it fails to create realistic content for removing massive objects from face images. Object removal techniques generally produce good results for small items with fixed locations, but they fail for massive objects with arbitrary locations.

Learning-based image editing approaches [20,21,22,23,24,25,26,27,28,29] outperform non-learning-based object removal methods quantitatively and qualitatively. There has been a significant amount of learning-based image editing work using the generative adversarial network that has been proposed. For example, Li et al. [20] suggested a GAN-based face completion method (GFCM). Compared to other approaches, this generative face completion method (GFCM) contains an extra global discriminator that verifies the realism of a produced face image and maintains the consistency of the whole face image. Although the GFCM can produce semantically acceptable results, it has a few flaws, such as the need for an image amalgamation operation to apply color coherency near the hole borders, and the reconstructed face image has some artifacts, mainly when the covered parts are near the image’s borders.

Iizuka et al. [21] suggested a globally and locally consistent image completion (GLCIC) method to complete a missing area in an image of any size. However, it has a lot of noise and artifacts in the recovered region, especially when there are holes towards the edges. GLCIC employs two discriminators combined with post-processing to make the produced component locally and globally consistent with the remainder of the image. GLCIC fills the image for random affected regions in face images. However, it is restricted to low resolutions (178 × 216), and it produces artifacts when the damaged area is towards the image’s edges. 

Yeh et al. [22] presented a semantic image completion approach based on a CGAN [23] on the known region to create the best uncorrupted image. Our technique determines the closest encoding and fills in the missing pixels by considering the context discriminator and the damaged image. The covered region has been effectively recovered, and the missing material has been well generated using this approach. The effects generated in the case of large missing regions are unreliable. Liao et al. [24] proposed a GAN-based collaborative adversarial learning method called Collaborative GAN (CollaGAN) for face recovery. This CollaGAN shows that a collaborative adversarial learning technique promotes direct face completion learning for improved semantic comprehension and, in turn, better face inpainting. The proposed CollaGAN model seeks to develop the face completion problem (e.g., landmark detection and semantic segmentation).

Yu et al. [25] offered a new GAN-based two-stage network for generative image in-painting that includes unique contextual attention (CA) layer that copies comparable feature patches from adjacent related visible regions to the missing regions. Although the entire network may be trained end-to-end, the copy–paste method may result in unwanted artifacts in the recovered portions. Song et al. [26] introduced the Geometry Aware Face Completion (GAFC) model, a two-stage network that performs a face completion job. A facial geometry estimator calculates the facial geometry of the face in the first phase. An encoder-decoder generator completes the face utilizing the facial geometry information in the second phase. Although the model outperformed many other face completion approaches, they come at a high computational cost due to the model’s prior knowledge of network extraction.

Nazeri et al. [27] presented a GAN-based Edge-Connect technique (EC) to recover the image after removing the unwanted objects. EC breaks the problem into two stages: edge generator and image completion. The image completion network completes the empty sections using hallucinated edges after the edge generator hallucinates the edges of the missing part. EC was able to restore the missing regions and achieve superior results. However, it cannot provide a realistic edge map in the event of large missing sections.

Din et al. [28] developed a GAN-based two-stage framework (MRGAN) to remove the medical face mask and reconstruct the mask-covered region. The first step detects the masks, and the reconstructed face is obtained in the second. The experimental results outperformed other image editing methods. This method, on the other hand, is complex and time-consuming. This approach also does not work well with various items (occluded face objects). Khan et al. [29] proposed a GAN-based use of a two-stage network for microphone removal. It produces plausible results when eliminating small objects, but unnatural results for big complex missing areas.

## 3. Our Approach

**Figure 2:**
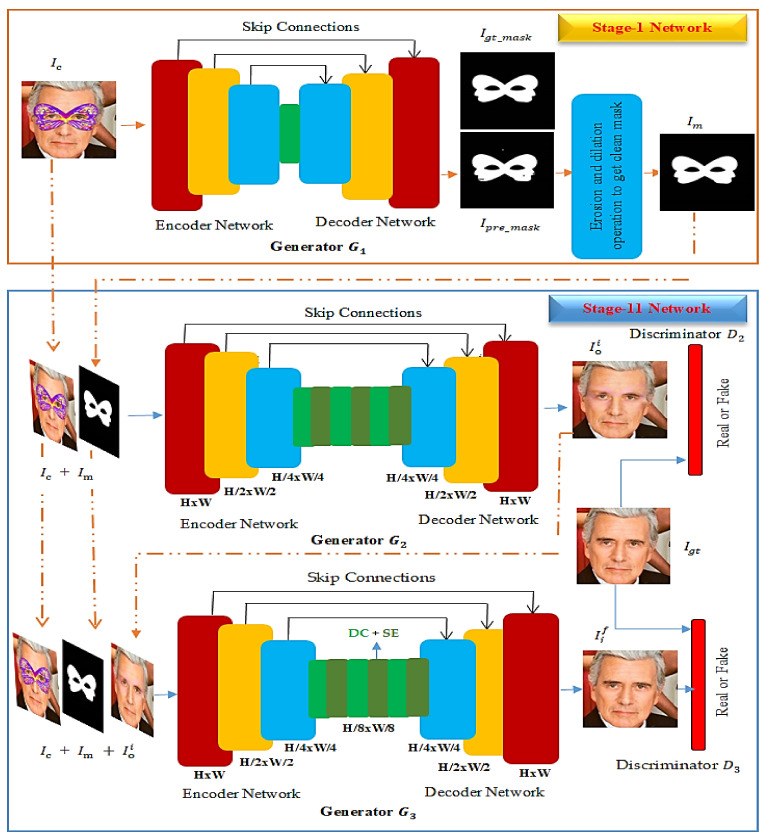
The architecture of the automatic mask removal network for face de-occlusion. It consists of Stage-I Network that generates a binary mask and Stage-II Network that removes the mask object from input facial images.

The general architecture of the proposed AFD-StackGAN is shown in Figure 2. Stage-I Network and Stage-II Network are the two major networks. The following sections consider each network in detail. Our task is to generate the binary mask simultaneously and remove the non-face object from the occluded image. Implementing this as an end-to-end model, we propose a two-stage approach to address this task. Each stage focuses on one aspect: Stage-I generates a binary mask, and Stage-II removes the mask object from the input facial image.

### 3.1. Stage-I Network: Binary Mask Generation Network

Stage-I Network (Binary Mask Generation Network) generates a binary mask after detecting the mask object in the input occluded facial image. The generator $G_{1}$ at Stage-I (Binary Mask Generation Network) takes the input image $l_{c}$ (occluded image) and generates a binary mask $l_{pre\_mask}$.

**Generator**$G_{1}$**.** The encoder of the generator $G_{1}$ takes the facial image $l_{c}$ as input and maps it to a low-dimensional latent representation (bottleneck layer). The decoder then maps back to a low-dimensional latent representation (bottleneck layer) to generate a binary mask $l_{pre\_mask}$ of the size of the input facial image. The architecture we design has three convolution layers for the encoder part and three convolutions (transpose convolution) layers for the decoder part, as shown in Figure 2. Each convolution layer is used in the form of a *relu + a convolution + a normalization layer*, except the first and last layers, which use a *tanh* in place of a *relu.* The decoder of $G_{1}$ is similar to the encoder, except that de-convolution layers substitute convolution layers. De-convolution layers are used in the decoder, gradually up-sampling latent representation to image scale. The decoder uses *tanh activation* without the normalization layer in the last layer.

**Loss Function.** $L_{l_{1}}$ loss is used to train Stage-I Network. The $L_{l_{1}}$ loss calculates the pixel-wise difference between a predicted binary mask $l_{pre\_mask}$ and target binary mask $I_{gt\_mask}$. $L_{l_{1}}$ loss is used to match the details of $l_{pre\_mask}$ with $I_{gt\_mask}$. The $L_{l_{1}}$ loss between $l_{pre\_mask}$ and $I_{gt\_mask}$ is expressed such as:

(1) $$L_{l_{1}}=\left|\left|l_{pre\_mask}-I_{gt\_mask}\right|\right|$$

where, $L_{l_{1}}$ loss is defined as the pixel-wise difference between a predicted binary mask $l_{pre\_mask}$ and target binary mask $l_{gt\_mask}$.

Binary masks $l_{pre\_mask}$ generated by $G_{1}$ are rough and have noise at some locations. To obtain a clean binary mask $l_{m}$, we utilized additional erosion and dilation morphological image processing techniques as a mask refiner network. Erosion removes salt noise from the generated mask $l_{pre\_mask}$ and dilution fills in the holes in the generated binary mask.

### 3.2. Stage-II Network: Face De-Occlusion Network

Stage-II Network (Face De-occlusion Network) aims to remove the occlusion mask from facial images and complete the region left behind with plausible content and fine details. Stage-II consists of a pair of generator and discriminator networks: $G_{2}\;$*+*$\;D_{2}$*, and* $G_{3}$*+*$D_{3}$. The generator $G_{2}$ takes the input occluded image $I_{c}$, along with the binary mask $I_{m}$, as a combined input and generates an occlusion-free image $I_{o}^{i}$. The generator $G_{3}$ takes the input image $I_{c}$, binary mask $I_{m}$, and $I_{o}^{i}$ (generator $G_{2}$ output) as a combined input and generates an occlusion-free final image $I_{i}^{f}$. The two discriminators $D_{2}$ and $D_{3}$, force generators $G_{2}$ and $G_{3}$ to produce visually plausible and naturalistic looking images by determining the $I_{o}^{i}$ (generator $G_{2}$ output) and $I_{i}^{f}$(generator $G_{3}$ output) as a real or fake face. The following sections consider each network in detail.

**Generator**$G_{2}$**.** Generator $G_{2}$ at Stage-II uses CNN-based encoding-decoding architecture. This encoder-decoder uses the idea of U-Net [11] with skip connections to prevent the loss of spatial information details at higher resolutions during the down-sampling and up-sampling functions of the encoder and decoder. The encoder takes the image $I_{o}$ as a concatenated input of occluded image $I_{c}$ (Stage-I input) and refine the binary mask $I_{m}$ (Stage-I output) and maps it to a low-dimensional latent representation. The decoder then maps back the low-dimensional latent representation, reconstructs and generates the initial coarse output facial image $I_{o}^{i}$. The encoder of $G_{2}$ is composed of five convolution layers (for simplicity, only three layers of the encoder are shown in Figure 2) progressively down-sampling the latent representation. Each convolution layer is used in the form of a *relu + a convolution + an instance normalization layer*, except the first and last layers, which use a *tanh* in place of a *relu*.

The decoder of $G_{2}$ is similar to the encoder, except that de-convolution layers substitute convolution layers. De-convolution layers are used in the decoder, gradually up-sampling the latent representation to image scale. A combination of dilated convolution (DC) [30] and Squeeze-and-Excitation (SE) blocks [31], as shown in Figure 2, is used in the middle of the encoder-decoder. DC is used to enhance the receptive field size without increasing the computational power and network parameters, making the recovered area under the occlusion mask convolutional network (FCN), which enhances a network’s representative power by learning the weights for more consistent with its surroundings. SE block is an addition to each feature map channel fully. SE-blocks recalibrate feature maps in the context of the channel.

**Discriminator**$D_{2}$. A PatchGAN discriminator $D_{2}$—which only penalizes structure at the scale of patches [32] and is used instead of regular GAN discriminators [1] to focus on reconstructing high-frequency content. Discriminator $D_{2}$ tries to decide if each patch of size 32 × 32 in an image $I_{o}^{i}$ (de-occluded image) is real or fake. We run $D_{2}$ convolutionally across the image $I_{o}^{i}$, averaging all responses to provide the ultimate output of $D_{2}$

**Loss Function.** To minimize the artifacts and ensure better visual quality, a careful arrangement (amalgam) of re-construction $L_{rc}$, perceptual $L_{per}$ And adversarial loss $L_{adv}$ (i.e., we unite re-construction loss, perceptual loss, and adversarial loss for each stage of Stage-II Network), is used to produce realistic and perceptually correct missing content occlusion-free face image. The joint loss function used to train the Stage-II Network (Face De-occlusion Network) is defined as:

(2) $$L_{joint}=\;\alpha L_{rc}+\beta L_{per}+L_{adv}$$

where *α* and *β* are constants to adjust the weights of re-construction loss and perceptual loss, respectively.

The re-construction loss composes of pixel-wise re-construction loss $L_{l_{1}}$ and structure-level similarity loss $L_{\mathrm{SSIM}}$. The re-construction loss can be written as:

(3) $$L_{rc}=L_{l_{1}}+L_{\mathrm{SSIM}}$$

The pixel-wise re-construction loss $L_{l_{1}}$ measure the per-pixel difference between generated occlusion-free face image $I_{o}^{i}$ and ground-truth $I_{gt}$. We calculate the pixel-wise re-construction loss via $l_{1}$-norm in place of $l_{2}$-norm because $l_{1}$-norm encourages less blurring and glaring errors than $l_{2}$-norm. The pixel-wise re-construction loss $L_{l_{1}}$ can be defined as

(4) $$L_{l_{1}}=\left|\left|I_{o}^{𝒾}-I_{gt}\right|\right|$$

where $\left|\left|\;.\;\right|\right|$ is the $l_{1}$-norm and $I_{o}^{𝒾}$ = $G_{2}$($I_{o}$) is the output image of the generator ($G_{2}$), i.e., face image without occlusion.

The structure-level similarity loss $L_{\mathrm{SSIM}}$ [33], which measures the structure-level difference between generated occlusion-free face image $I_{o}^{i}$ and ground-truth $I_{gt}$, can be defined as:

(5) $$L_{\mathrm{SSIM}}=1-\mathrm{SSIM}\;\left(I_{o}^{𝒾}\;,\;I_{gt}\right)$$

The perceptual loss $L_{per}$ which boosts the generator’s output to have identical representation to the ground truth measures the feature-level difference between the feature maps of the generated occlusion-free face image $I_{o}^{i}$ and ground truth $I_{gt}$, extracted by a VGG-19 network [34], which is pre-trained on ImageNet [35]. Let $\varphi_{𝒾}$ be the activation map of the 𝒾^th^ layer of the VGG-19 network, then the feature matching loss is defined as:

(6) $$\;\;\;L_{p}=\Sigma ||\varphi_{𝒾}\left(I_{o}^{𝒾}\right)-\varphi_{𝒾}(I_{gt\;})||$$

We exploit the intermediate convolution layer feature maps (conv_3, conv_4 and conv_5) of the VGG-19 network to obtain rich structural information, which helps in recovering a plausible structure for the face semantics.

In addition to re-construction loss $L_{rc}$, and perceptual loss $L_{per}$, the adversarial loss $L_{adv}$, used to render the repaired image $I_{o}^{𝒾}$ as real as possible and generate realistic results, can be expressed in Equation (7).

(7) $$L_{\mathrm{adv}}=\begin{array}{c} \min\\ G_{2} \end{array}\begin{array}{c} \max\\ D_{2} \end{array}E[\log(D_{2}(I_{o}^{𝒾},\;\;I_{gt}))]+[\log(1-D_{2}(G_{2}\left(I_{o})))\right]$$

where $\;I_{gt}$ represents the real sample (ground-truth), $I_{o}^{𝒾}$ represents the initially generated de-occluded image, $I_{o}$ is the concatenated input for $G_{2}$, E represents the expectation, and $L_{\mathrm{adv}}$ represents the adversarial loss at the base network. The $\log\left(D_{2}\left(I_{o}^{𝒾},\;\;I_{gt}\right)\right)$ is the loss function for $D_{2}$ and $\log\left(1-D_{2}\left(G_{2}\;\left(I_{o}\right)\right)\right)$ is the loss function for $G_{2}$.

**Generator**$G_{3}$**.** Generator $G_{3}$ at Stage-II is quite similar to the generator $G_{2}$. We propose $G_{3}$ to bring the initial result $I_{o}^{i}$ ($G_{2}$ result) closer to the ground truth by rectifying what is missing or wrong in the initial result. To achieve this, we feed $I_{c}$ and $I_{m}$ ($G_{2}$ inputs) again with $I_{o}^{i}$ ($G_{2}$ output) as a concatenated input $I_{0}^{f}$ into $G_{3}$, which generates the final result $I_{i}^{f}$ with more photorealistic details in the recovered area. We feed $I_{o}$ and $I_{m}$ ($G_{2}$ inputs) again to enforce edge consistency at the affected region boundary, further increasing the generated face image’s visual quality.

**Discriminator**$D_{3}$**.** A Patch-GAN discriminator $D_{3}$ at Stage-II shares the identical architecture as $D_{2}$. Discriminator $D_{3}$ tries to classify if each patch of size 32 × 32 in an image $I_{i}^{f}$ (final de-occluded image) is real or fake. We run this discriminator $D_{3}$ convolutionally across the image $I_{i}^{f}$, averaging all responses to provide the ultimate output of $D_{3}$.

**Loss Function.** Note: We incorporate the same re-construction loss $L_{\mathrm{rc}}$, and perceptual loss $L_{\mathrm{per}}$ to produce a final de-occluded image. Thus, we do not mention them separately. The adversarial loss $L_{\mathrm{adv}}$ is used to make the repaired image $I_{i}^{f}$ as real as possible and generated realistic results, which can be expressed in Equation (8).

(8) $$L_{adv}=\begin{array}{c} \min\\ G_{3} \end{array}\begin{array}{c} \max\\ D_{3} \end{array}E[\log(D_{2}(I_{𝒾}^{f},\;\;I_{gt}))]+[\log(1-D_{3}(G_{3}\left(I_{0}^{f})))\right]$$

where $\;I_{gt}$ represents the real sample (ground-truth), $I_{i}^{f}$ represents the finally generated de-occluded image, $I_{0}^{f}$ is the concatenated input for $G_{3}$, E represents the expectation, and $L_{adv}$ represents the adversarial loss at the refiner network. The $\log\;\left(D_{3}\left(I_{i}^{f},\;\;I_{gt}\right)\right)$ is the loss function for $D_{3}$ and $\log\left(1-D_{3}\left(G_{3}\;\left(I_{0}^{f}\right)\right)\right)$ is the loss function for $G_{3}$.

### 3.3. Total Loss Function

The total loss function used to train the whole module is a weighted sum of $L_{l_{1}}$ (Equation (1)) and $L_{joint}$ (Equation (2)), defined as:

(9) $$L_{\mathrm{total}}=L_{l_{1}}+\alpha L_{\mathrm{rc}}+\beta L_{\mathrm{per}}+L_{\mathrm{adv}}$$

where α and β are the constants for altering the weights of reconstruction and perceptual loss. For the first part of Stage-II ($G_{2}+D_{2}$), we used α = 100 and 𝛽 = 33 to capture better structure, and for the second part of Stage-II ($G_{3}+D_{3}$), we used α = 10 and 𝛽 = 3.3 for yielding natural-looking results.

## 4. Experiments

In this section, firstly, we describe the training and implementation details of the proposed approach. Afterward, we introduce the competing baseline models. Finally, this section explains the synthetic dataset creation used for training and the real-world dataset used for evaluation.

### 4.1. Training and Implementation Details

For training of Stage-I Network, we input facial images $I_{c}$ into mask generation network, which generates a binary mask $I_{pre\_mask}$ close to the target binary mask $I_{gt\_mask}$. $I_{pre\_mask}$ is then fed into a mask object refiner network and generates a final binary mask $I_{m}$. For training of Stage-II Network, we input facial images $I_{c}$ (input of Stage-I) and binary mask, $I_{m}$ (output of Stage-I), and generate an occlusion-free facial image $I_{o}^{i}$. Then, $I_{o}^{i}$ (Initially generated de-occluded image), $I_{c}$ (input of Stage-I), and binary mask, $I_{m}$ (output of Stage-I), are fed into an image refiner network ($G_{3}$) that produces a final occlusion-free facial image $I_{i}^{f}$.

TensorFlow [36] is used to implement the proposed model and is trained with Nvidia GTX 1080Ti GPU. We trained the proposed model with batch size 10 and utilized Adam [37]. The model was trained for 1000 iterations. We used TTUR [38] for training. The learning rate of 0.0001 for the generator and 0.0004 for the discriminator in both stages were employed. GAN training becomes more stable using different learning rates for generator and discriminator updates.

### 4.2. Competing Methods

After reviewing various related approaches in Section 2, GLCIC (Iizuka et al. [21]), GCA (Yu et al. [25]), EdgeConnect (Nazeri et al. [27]), and MRGAN (Din et al. [28]) are the closest approaches to our work. MRGAN is a GAN-based two-stage framework for removing amedical face mask and reconstructing the mask-covered region. While impressive results were produced in removing medical masks, their network is incapable of automatically detecting and removing multiple types of complex objects. In contrast, the proposed model (AFD-StackGAN) can automatically detect and remove multiple complex objects of various sizes, shapes, colors, and structures. EdgeConnect also uses a two-stage adversarial approach in which it generates the guidance information in the first stage and edits the image in the second stage. It successfully recovers the image based on hallucinated edge information from an edge generator network. Unlike EdgeConnect, the proposed model generates a binary mask of the non-face object (i.e., masked region) while EdgeConnect generates the edge map of the complete image. Moreover, it uses a GAN setup with one discriminator in both stages while the proposed model employs two separate discriminators in both stages with two separate generators, which uses CNN-based encoding-decoding network architecture with Skip-connection, which is used in the generator network to strengthen the predictive ability of the generator and to prevent the gradient vanishing caused by the deep network. The result shows that the image completed by the encoder-decoder network architecture with Skip-connection is more realistic.

In contrast, GLCIC and GCA train both discriminators jointly at the same time along with one generator to learn global consistency and deep missing region with a post-processing step such as poison image blending, while we train both discriminators along with two separate generators and our work does not use any supplementary processing or post-processing step. GLCIC and GCA models have noticeable artifacts and blurriness in the generated regions since these models predict the missing regions from only high-level features. Different from GLCIC and GCA, the proposed model predicts the missing regions from both low-level and high-level features (pixel-wise loss ($l_{1}$) for low-level features and Structural Similarity loss (SSIM) for high-level features). These schemes are not suitable for our problem because they cannot overcome the complexity of the task and produce artifacts due to large missing regions of arbitrary shape.

### 4.3. Datasets

#### 4.3.1. Synthetic Generated Dataset

**Figure 3:**
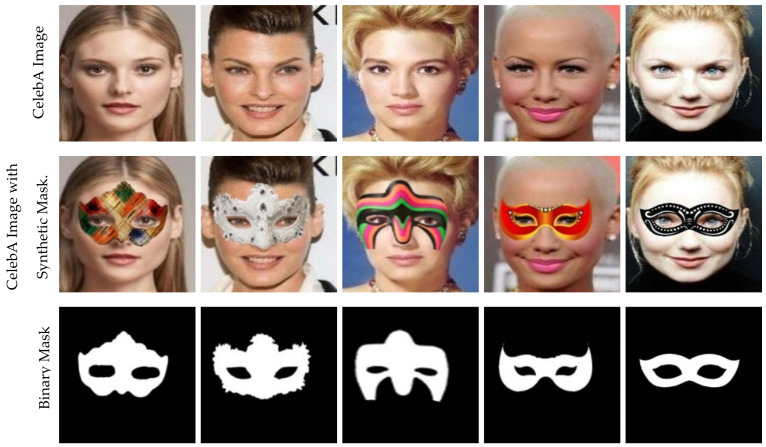
Some images of our synthetic dataset.

For supervised training of our model, no publicly accessible dataset comprises face image pairings with or without mask objects. We have created a synthetic dataset using the publicly available CelebA Face dataset [39]. With more than 200k celebrity images, CelebA is a vast face attribute collection. To create synthetic samples, we randomly place mask objects of various sizes, shapes, colors, and structures in the images using Adobe Photoshop CC 2018, as shown in row two of Figure 3. Then, we create the binary masks of the corresponding mask objects, as shown in row three. All input images and masks in our synthetic dataset have a resolution of 256 × 256. Figure 3 shows some sample images of our synthetic dataset. Further descriptions of our synthetic dataset are given in Table 1. 

#### 4.3.2. Real-World Generated Dataset

A dataset of occluded facial images downloaded from the Internet was formed to demonstrate the proposed method’s effectiveness on real-world data. While creating these occluded facial images dataset, we took all possible care to ensure that the images collected from the Internet were diverse in sizes, shapes, structures, and positions regarding the occlusion masks. Additionally, the binary mask of the corresponding occluded region for real-world data using Adobe Photoshop 2018 was developed, since manually generated binary masks for the occluded region are provided with input occluded facial images at training and inference stages. This dataset is used for evaluation (test) purposes only. Each image in real-world data has a resolution of 256 × 256.

### 4.4. Performance Evaluation Metrics

Although the GAN-based models have achieved great success in numerous computer vision applications, it is still difficult to evaluate which methods are better than other methods because there is no standard defined function for quantitative evaluation, which hurts the GAN performance. Nevertheless, to quantitatively and objectively analyze the accuracy or effectiveness of the proposed system, various numerical evaluation metrics are chosen, such as Structural Similarity (SSIM) [33], which guesses the all-inclusive similarity between the reconstructed and the target face images, Peak Signal-to-Noise Ratio (PSNR) is one of the most widely used full-reference quality metrics that measure the difference in pixel values between the reconstructed and the target face images, Mean Square Error (MSE) calculates the average squared difference between the reconstructed and the target face images, Naturalness Image Quality Evaluator (NIQE) [40], which measures the quality of image, and Blind/Referenceless Image Spatial Quality Evaluator (BRISQUE) [41], which calculates naturalness of image.

Greater PSNR and SSIM values mean closer distances between synthetic data and real data distributions (i.e., greater PSNR and SSIM values show good performance of the generative model). In comparison, lower PSNR and SSIM values indicate greater distances between synthetic data and real data distributions (i.e., lower PSNR and SSIM values show the generative model’s bad performance). Lower MSE, NIQE, and BRISQUE values mean closer distances between synthetic data and real data distributions (i.e., lower MSE, NIQE, and BRISQUE values show good performance of the generative model). In comparison, higher MSE, NIQE, and BRISQUE values mean greater distances between synthetic data and real data distributions (i.e., higher MSE, NIQE, and BRISQUE values show the poor performance of the generative model).

## 5. Results and Comparisons

We designed an automatic mask generation network for face de-occlusion to remove mask objects. This automatic mask generation network automatically detects mask objects, generates binary masks, and then removes the masks objects. This section covers the results of Stage-I Network, Stage-II Network. We also discuss and compare the qualitative and quantitative performance of the proposed model with baseline models. 

### 5.1. Results of Stage-I Network

**Figure 4:**
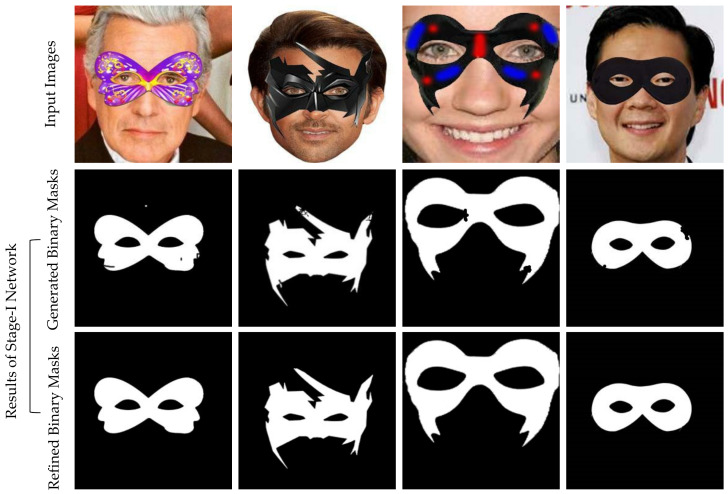
The results of Stage-I Network on real-world images.

Figure 4 shows the results of the Stage-I Network on real-world images. The first row contains input images with mask objects. The mask generation network successfully generated binary masks, as listed in the second row. The third row displays the results of the mask refiner network, which improves the results by rectifying what is wrong or missing in the mask generator network results. Finally, these masks are used as input to Stage-II Network.

### 5.2. Results of Stage-II Network

**Figure 5:**
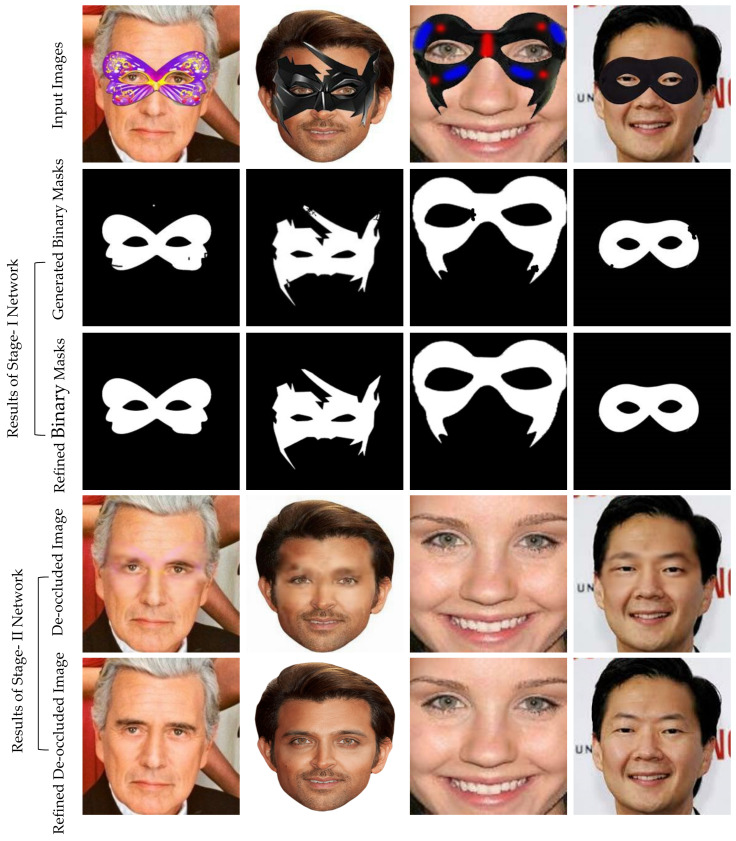
The results of AFD-StackGAN (Stage-I Network + Stage-II Network) on real-world images.

Figure 5 shows the results of Stage-II Network on real-world images. The first row contains input images, the second row features corresponding binary masks generated by the mask generation network, the third row contains refined mask refined by the mask refiner network, and the last two rows show the output of Stage-II Network (Face De-occlusion Network). It can be seen that the proposed Face De-occlusion Network successfully generates correct face semantic structure and texture without any interaction. Therefore, this fully automatic approach can be used for practical implementation, such as live video.

### 5.3. Qualitative Comparisons

**Figure 6:**
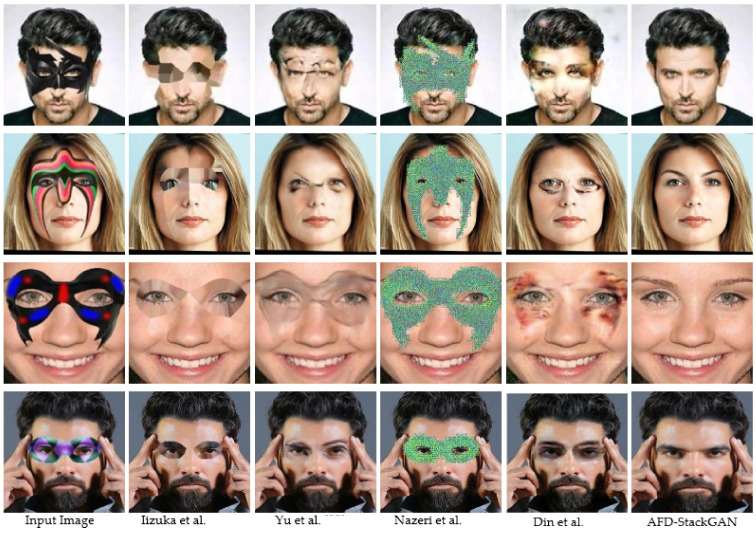
Visual assessment of the proposed AFD-StackGAN with the baseline models on real-world images.

The sample quality is primarily evaluated based on the visual fidelity generated by the GAN-based frameworks in the absence of a consistent and robust assessment method. Figure 6 shows the results of the proposed AFD-StackGAN and the baseline models (Iizuka et al. [21], Yu et al. [25], Nazeri et al. [27], and Din et al. [28]) on real-world images. We showed the input facial images and the output occlusion-free facial images in the qualitative experiments’ test set. For this, we give the qualitative results of the proposed AFD-StackGAN and baseline models. It can be seen in Figure 6 that the results of the proposed AFD-StackGAN are smoother and more realistic than the baselines models’ generated results for real data. Quantitative results show that the proposed AFD-StackGAN can handle occluded facial images under challenging conditions, e.g., complex occlusions with variations in size, structure, type, shape, and position in the facial image.

**Figure 7:**
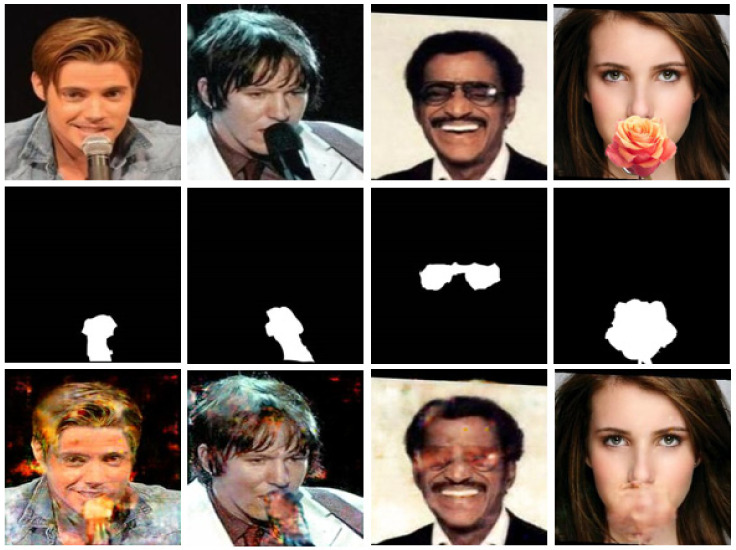
AFD-StackGAN performance for real face images with occlusion masks that have very different structures and locations in the face images than the occlusion masks used in the synthetic dataset. The first row shows occluded input facial images, and the second row shows de-occluded output face images.

- **Hard Examples.** Although the proposed AFD-StackGAN can handle the removal of occlusion masks of various shapes, sizes, colors, and structures, even on images not used to train the network, there are some examples, as shown in Figure 7, AFD-StackGAN fails to remove the occlusion masks altogether. Common failure cases occur when the Stage-I Network (Binary Mask Generation Network) cannot produce a good binary mask of the mask object, as shown in the first row of Figure 7, failing to detect them correctly. This happened when occlusion masks were different from those in our synthetic dataset in shape, position, and structure, as they mainly cover the regions around both eyes. As seen in the first row of Figure 7, the mask objects’ shapes, colors, positions, and structures are different from the mask types we used in our synthetic dataset. Moreover, the proposed model was trained using images from the CelebA dataset, and the CelebA data set images are roughly cropped and aligned, while the other dataset image (e.g., real-world images) are not processed in this manner, as shown in the first row of Figure 7. Our model cannot handle unaligned faces well and fails to generate missing regions of the images with unaligned faces. As expected, AFD-StackGAN produces worse results overall, as seen in the third row.

### 5.4. Quantitative Comparisons

To quantitatively compare the performance between the proposed model and the baseline models, we use the following five performance evaluation metrics: (1) SSIM, (2) PSNR, (3) MSE, (4) NIQE, and BRISQUE (as explained in Section 4.4). The quantitative score via SSIM, PSNR, and MSE is evaluated using the synthetic test dataset results because no ground truth exists for real occluded face images since they were downloaded from the Internet, while the quantitative score via NIQE and BRISQE is evaluated using the results from the real test samples. For MSE, NIQE, and BRISQUE, smaller values indicate superior efficiency, while for PSNR and SSIM, the higher, the better. The quantitative scores in terms of SSIM, PSNR, MSE, NIQE, and BRISQUE of proposed AFD-StackGAN and baseline models are shown in Table 2. Table 2 shows the averaged test scores obtained from individual test images. It has been observed that AFD-StackGAN generates semantically consistent and visually plausible face images without occlusion masks, which can help improve the performance of many computer vision algorithms for face identification/recognition purposes in future studies.

### 5.5. Ablation Studies

This section presents the ablation studies to understand the usefulness of using an automatically generated mask than a manually generated mask and the role of using the refiner networks in both stages.

#### 5.5.1. Performance Comparison between Using User-Defined Mask and Auto-Defined Mask

**Figure 8:**
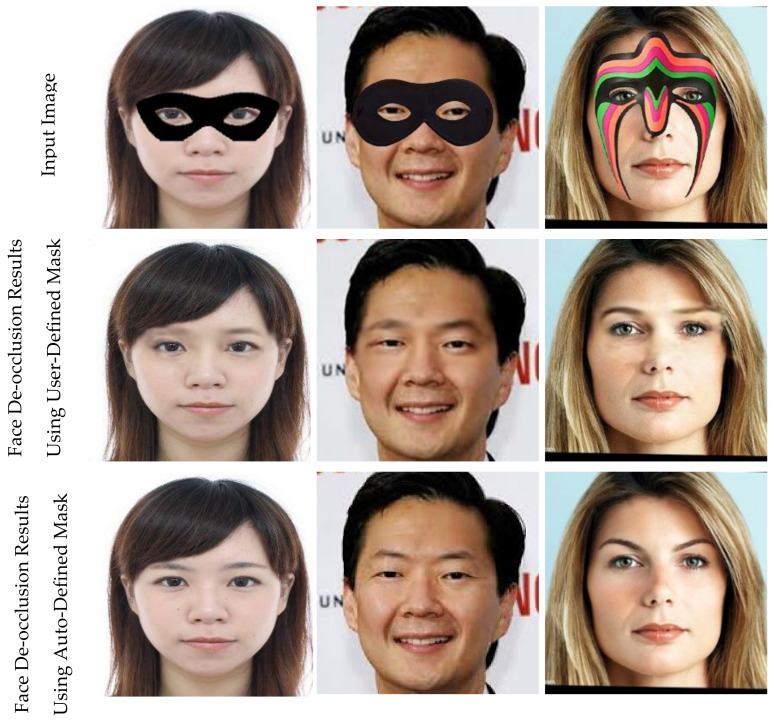
Visual comparison of the automatic mask removal network (used auto-generated mask) with FD-StackGAN (used user-defined mask).

To evaluate the effectiveness of the proposed method, we compared the performance between directly using the user-defined manually generated binary mask and automatically generated binary mask. The first column in Figure 8 contains the input images. The second column in Figure 8 is the editing result by using a user-defined manually generated binary mask. The third column represents the editing results obtained using an automatically generated binary mask. We can see that the editing result by using an automatically generated binary mask is better than using a user-defined manually generated binary mask. Table 3 shows the quantitative scores of the proposed method with a user-defined mask and auto-defined mask

Note that the editing result using the user-defined manually generated binary mask is obtained by only running Stage-II Network without Stage-I Network. The user-defined manually generated binary mask inputs Stage-II Network and the input image.

#### 5.5.2. Role of Refiner Networks

**Figure 9:**
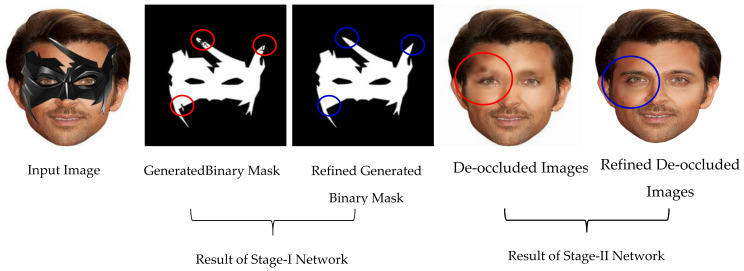
Results of image refiner network on real-world images further improve the results by rectifying what is missing or wrong in the mask base network results.

We performed the ablation study to show the effectiveness of refiner networks in the proposed multi-stage approach. For this, we drew a qualitative comparison by training the proposed model with a refiner network and without a refiner network. As shown in Figure 9, each stage of the proposed model trained with the refiner network can generate more photorealistic results with minimum-deformation artifact-free images than the results of each stage of the proposed model trained without the refiner network.

In the first stage of our model, the mask generation network generates a binary mask automatically. The mask generation network-generated results (i.e., binary mask) have some noise at some locations (red circles are used to specify the locations of some noise artifacts). The refiner network removes the noise in the mask generation network-generated results (blue circles specify the areas and locations of some refinement corrections). Stage-I Network can generate a more noise-free binary mask with the help of a refiner network.

In the second stage of our model, the face de-occluded network removes the mask object and completes the area left behind with plausible content and fine details. The initially generated results are generally blurry with missing details and several defects, especially for masked areas (red circles are used to specify the locations of some undesired artifacts). The refiner network corrects what is missing or wrong in the initially generated results (blue circles are used to specify the areas and locations of some refinement corrections) and generated results that contain more photorealistic details with minimum undesired artifacts. Stage-II Network can generate more natural-looking images with the help of a refiner network.

## 6. Conclusions

This work proposed a two-stage GAN-based model that successfully recovers the de-occluded facial image after automatically generating the mask of the non-face object in the occluded input facial image. Previous approaches cannot resolve well issues related to removing numerous mask objects covering large discriminative regions of the person’s face. In contrast, the proposed model can successfully remove the numerous types of mask objects of large complex nature in the facial images, covering most of the person’s face by creating semantically applicable and visually plausible content for the missing regions. The performance on real world data is quite satisfactory although we train our network using the synthetic dataset only. We analyze the proposed model performance quantitatively and qualitatively and show that the proposed model can produce structurally consistent results of higher perceptual quality. The proposed model is quite flexible to handle vast missing regions or covered regions that vary in structures, sizes, colors, and shapes.

Since AFD-StackGAN is trained on a synthetic dataset, there could be a domain discrepancy between real-world test facial images and synthetic training facial images. To manage this issue, domain adaptation would be required to reduce the domain distance between real images and synthetic ones, potentially solving the problem. We have planned to work in this domain to settle this issue in the future.

## Figures and Tables

**Table 1:** A summary of dataset feature description used in experiments.

Synthetic Generated Dataset	Feature Description
Total Number of Samples	20,000
Number of Training Samples	18,000
Number of Testing Samples	2000
No. of Classes	50
Samples Per Class	400
Number of Training Samples	18,000

Note: In the above table, number of classes indicates how many mask objects (non-face objects) varied in sizes, shapes, structures, and positions are used in the synthetic generated dataset, and samples per class indicates on how many images (faces) a specific mask object is applied.

**Table 2:** Performance comparison of different methods in terms of SSIM, MSE, PSNR, NIQE, and BRISQUE. For PSNR and SSIM, higher values show superior performance, while for BRISQUE and NIQE, the lower, the better.

Methods	SSIM ↑	PSNR ↑	MSE ↓	NIQE ↓	BRISQUE ↓
Iizuka et al. [21]	0.763	21.953	2329.062	4.754	34.106
Yu et al. [25]	0.797	15.469	2316.839	4.951	32.761
Nazeri et. [27]	0.561	15.848	2450.889	16.991	36.426
Din et al. [28]	0.850	16.209	2223. 938	5.721	31.016
AFD-StackGAN	0.978	33.201	32.435	4.902	39.872

**Table 3:** Performance comparison between using user-defined mask and auto-defined mask in SSIM, PSNR, MSE, NIQE, and BRISQUE.

Methods	SSIM ↑	PSNR ↑	MSE ↓	NIQE ↓	BRISQUE ↓
User-Defined Mask	0.981	32.803	34.145	4.499	42.504
Auto-Defined Mask	0.978	33.201	32.435	4.902	39.872

## Data Availability

Not applicable.

## Abbreviations

GAN	Generative Adversarial Network
CNN	Convolutional Neural Network
FCN	Fully Convolutional Network
SE	Squeeze and Excitation block
DC	Dilated Convolution
TTUR	Two Time-scale Update Rules
**Notations**	
$l_{c}$	Occluded image
$l_{gt}$	Ground truth image
$l_{pre\_mask}$	Generated binary mask
$l_{m}$	Noise-free binary mask
$l_{c}$	$\mathrm{Concatenated}\;\mathrm{input}\;\mathrm{of}\;\mathrm{occluded}\;\mathrm{image}\;l_{c}\;$ $\mathrm{and}\;\mathrm{generated}\;\mathrm{binary}\;\mathrm{mask}\;l_{pre\_mask}$
$I_{o}^{i}$	Initially generated de-occluded facial image
$I_{o}^{f}$	$\mathrm{Concatenated}\;\mathrm{input}\;\mathrm{of}\;\mathrm{occluded}\;\mathrm{image}\;l_{c}$ $,\;\mathrm{generated}\;\mathrm{binary}\;\mathrm{mask}\;l_{pre\_mask}\;\mathrm{and}$ $\;\mathrm{initially}\;\mathrm{generated}\;\mathrm{de}-\mathrm{occluded}\;\mathrm{facial}\;\mathrm{image}\;I_{o}^{i}$
$I_{i}^{f}$	Finally generated de-occluded facial image
		
